# A Novel Case of Resolved Postherpetic Neuralgia with Subsequent Development of Trigeminal Neuralgia: A Case Report and Review of the Literature

**DOI:** 10.1155/2013/398513

**Published:** 2013-07-30

**Authors:** Alexander Mason, Kristen Ayres, Sigita Burneikiene, Alan T. Villavicencio, E. Lee Nelson, Sharad Rajpal

**Affiliations:** ^1^Boulder Neurosurgical Associates, 4743 Arapahoe Avenue, Suite 202, Boulder, CO 80303, USA; ^2^Justin Parker Neurological Institute, Boulder, CO 80303, USA

## Abstract

A 72-year-old female patient is presented, who was diagnosed with herpes zoster along the left ophthalmic branch of the trigeminal nerve with associated cutaneous vesicles. The patient subsequently developed postherpetic neuralgia in the same dermatome, which, after remission, transformed into paroxysmal trigeminal pain. The two different symptom sets, with the former consistent with PHN and the later consistent with trigeminal neuralgia, were unique to our practice and the literature.

## 1. Introduction

 Herpes zoster (HZ) is classified as the reactivation of the varicella-zoster virus (VZV) and is commonly known as shingles. HZ causes an inflammatory immune response, which affects the ganglia of the sensory nerves [1]. While often self-limiting, in an estimated 10% of patients this can lead to chronic pain in the affected dermatome(s) known as postherpetic neuralgia (PHN) [2], which is characterized by constant variable symptoms including burning pain in the affected dermatome [1].

 Discretely different pathophysiologically and affecting the trigeminal dermatomes, trigeminal neuralgia (TN) is a neuropathic pain disorder characterized symptomatically by a sudden and severe stabbing pain. It is classically associated with microvascular compression of the trigeminal nerve but other etiologies have been suggested and are discussed below [3].

 This report examines a novel case of a patient who is experiencing both PHN and TN, the first reported in the literature. 

## 2. Case Report

 Seventy-two-year-old female diagnosed with left ophthalmic branch of the trigeminal nerve (V1) HZ with associated cutaneous vesicles. This was self-limited in its course, although she then subsequently developed PHN in the same dermatome, characterized by a burning pain that was constant. This constant burning paraesthesia in her left V1 persisted for approximately five years and gradually improved. Then, without an inciting event, approximately 12 months prior to presentation in our office, she was experiencing a paroxysmal, lancing, sharp and painful, stabbing-like pain in the V1 dermatome that was triggered by eating, brushing teeth, and occasionally yawning. A brain MRI was done to rule out multiple sclerosis or structural abnormality. The two different symptom sets, with the former consistent with PHN and the later consistent with TN, were unique to our practice and the literature. 

## 3. Discussion

 Herpes zoster affects approximately 4 patients/1,000 Americans per year. Fifty-six percent of patients have thoracic dermatomes affected; up to 25% of patients have cranial trigeminal nerve involvement, most commonly the first division [4, 5]. Pathophysiologically in HZ, varicella zoster virus can become latent, typically in the neuronal cell bodies. Years or decades later, the virus may become activated and migrate distally along the axon, leading to cutaneous manifestation of the infection of HZ. Postherpetic neuralgia can occur with injury secondary to altered gene expression in the sensory nerves that can induce neurochemical, physiological, and/or anatomical modifications (such as afferent terminal sprouting) [6, 7]. This can lead to a hyperexcitable state of the neuron that is associated with PHN [8]. The immune system responds to the activation of the virus and produces anti-inflammatory cytokines and chemokines, which can further damage the nerves of the dorsal root ganglia. These molecules can also cause pain by directly influencing the activity of nociceptors [8]. It is estimated that 10% of HZ patients develop PHN [1]. Risk factors for PHN include advanced age, greater acute pain, severe rash, prodromal pain, ophthalmic location, and possibly female sex [1]. By the age of 60 years old, the risk increases to 60% [2]. The incidence of HZ patients who develop PHN also varies depending on the definition of the condition [1].

 Trigeminal neuralgia was found to have an incidence of 2–5/100,000/year, making it much less common than PHN [2]. Like PHN, risk for TN increases with age. Incidence was found to increase to 17.5/100,000/year for ages 60–69 and 25.6/100,000/year after age 70 [2]. Although there are many idiopathic cases of TN, it has been found to be comorbid with such disorders as arterial hypertension, Charcot-Marie-Tooth neuropathy, and glossopharyngeal neuralgia [2]. Other, more common, etiologies include vascular compression of the trigeminal nerve and multiple sclerosis (2%–4% of cases) [3]. Trigeminal neuralgia is caused most commonly by microvascular compression of the trigeminal root at the root entry zone, which is thought to cause demyelination at that location. The mechanism is thought to be neuronal depolarization leading to over production of action potentials, resulting in nonevoked pain responses [9]. Multiple sclerosis is also a well-studied etiology of TN [10].

 Postherpetic neuralgia has been classified into three subtypes; irritable nociceptor with allodynia, deafferentation with allodynia, and deafferentation without allodynia. Irritable nociceptor with allodynia is characterized by severe pain, mechanical allodynia, and minimal loss of heat sensation [11, 12]. Deafferentation either with or without allodynia differs in that patients have deficits in thermal sensation [11, 12]. Although patients with typical TN may have allodynia, but this disorder is characterized by sudden attacks of sharp, stabbing pain induced by mechanical, chemical, and thermal stimuli and lasting only a few seconds to a few minutes [13]. Atypical TN is also characterized by these paroxysmal pains, but includes a constant pain as well; other variants are also reported [13].

 Although allodynia due to PHN and TN are similar in the clinical view, the distinction between the two comes from their natural histories [14]. Postherpetic neuralgia is a neuropathic pain that has a clear origin from the infection with the herpes zoster virus [14]. Trigeminal neuralgia has a spontaneous onset and has an idiopathic nature [14]. A study by Attal et al. found that typical TN was consistent with electric shock like pain only, while PHN patients typically experienced allodynia and burning pain [15]. This suggests that although certain subtypes of PHN and TN are clinically similar, these two conditions can occur independently and that a single patient can present with both PHN and TN, as seen in this case.

 The patient in this case report experienced distinct cases of PHN and TN. She initially developed the characteristic constant burning pain associated with PHN, which then essentially resolved. She then later developed the sudden stabbing pain by the triggers noted above, a pain more clearly associated with TN. There seem to be no other reported cases of patients experiencing these distinct consecutive conditions. The consideration of a reactivation of HZ, with development of a different subtype of postherpetic neuralgia was considered, although the patient denied any of the symptoms typically associated with HZ with the appearance of the secondary pain; additionally, the symptoms of the secondary pain syndrome were identical to that of TN. Alternatively, one could hypothesize that the irritated or damaged sensory nerve from the PHN may have been predisposed to the microvascular insult classically associated with TN. Regardless of the mechanism, this novel case reminds the clinician to be cognizant of the nuances and potential overlap of postherpetic and trigeminal neuralgias without any pathophysiological links between the two diseases. 
